# MaSln1, a Conserved Histidine Protein Kinase, Contributes to Conidiation Pattern Shift Independent of the MAPK Pathway in *Metarhizium acridum*

**DOI:** 10.1128/spectrum.02051-21

**Published:** 2022-03-28

**Authors:** Zhiqiong Wen, Yuxian Xia, Kai Jin

**Affiliations:** a Genetic Engineering Research Center, School of Life Sciences, Chongqing University, Chongqing, People’s Republic of China; b Chongqing Engineering Research Center for Fungal Insecticide, Chongqing, People’s Republic of China; c Key Laboratory of Gene Function and Regulation Technologies, Chongqing Municipal Education Commission, Chongqing, People’s Republic of China; Broad Institute

**Keywords:** entomopathogenic fungus, Sln1, virulence, conidiation pattern shift, MAPK pathway

## Abstract

As a conserved sensor kinase in the HOG-MAPK pathway, Sln1 plays distinct functions in different fungi. In this study, the roles of MaSln1 in Metarhizium acridum were analyzed using gene knockout and rescue strategies. Deletion of *MaSln1* did not affect conidial germination, conidial yield, or resistance to chemical agents. However, fungal tolerance to heat shock and UV-B were significantly reduced after deletion of *MaSln1*. Insect bioassays showed that fungal pathogenicity was significantly impaired when *MaSln1* was deleted. Further studies showed that MaSln1 did not affect either germination or appressorium formation of *M. acridum* on locust wings, but it significantly increased appressorium turgor pressure. In addition, disruption of *MaSln1* resulted in a conidiation pattern shift in *M. acridum*. Microscopic observation revealed, however, that some genes located in the MAPK signaling pathway, including *MaSho1*, *MaHog1*, *MaMk1*, and *MaSlt2*, were not involved in the conidiation pattern shift on SYA medium (microcycle medium). Meanwhile, of the 143 differently expressed genes (DEGs) identified by RNA-seq, no genes related to the MAPK pathway were found, suggesting that MaSln1 regulation of the conidiation pattern shift was probably independent of the conserved MAPK signaling pathway. It was found that 22 of the 98 known DEGs regulated by MaSln1 were involved in mycelial growth, cell division, and cytoskeleton formation, indicating that MaSln1 likely regulates the expression of genes related to cell division and morphogenesis, thus regulating the conidiation pattern shift in *M. acridum*.

**IMPORTANCE** The productivity and quality of conidia are both crucial for mycopesticides. In this study, we systematically analyzed the roles of *MaSln1* in fungal pathogens. Most importantly, our results revealed that deletion of *MaSln1* resulted in a conidiation pattern shift in *M. acridum*. However, some other genes, located in the MAPK signaling pathway, were not involved in the conidiation pattern shift. RNA-seq revealed no genes related to the MAPK pathway, suggesting that the regulation of the conidiation pattern shift by MaSln1 was probably independent of the conserved MAPK signaling pathway. This study provided a new insight into the functions of Sln1 and laid a foundation for exploring the mechanisms of conidiation pattern shifts in *M. acridum*.

## INTRODUCTION

Entomopathogenic fungi can penetrate the host cuticle and utilize the nutrition in the hemocoel (1), and have attracted lots of attention for arthropod pest control (2, 3). Conidia play critical roles in the process of infecting their hosts, surviving in the environment, and spreading in the pest population (4–6). Most filamentous fungi have two conidiation patterns, microcycle conidiation and normal conidiation (7). In normal conidiation, conidia are generated after the mycelia have properly extended, while in microcycle conidiation, new conidia can be formed directly from the germinated ones (8). Under certain, specific conditions, the two patterns are interconvertible (9–11). Two conidiation patterns also exist in Metarhizium acridum, a locust-specific pathogen, and microcycle conidiation exhibited great potential for improving the productivity and quality of conidia (12). It is therefore important to clarify the underlying mechanisms of conidiation pattern shifts in entomopathogenic fungi.

The mitogen-activated protein kinase (MAPK) signaling pathway plays important roles in fungal growth and development, such as cell cycle, morphogenesis, stress resistance, virulence, cell wall rigidity and integrity, intercellular signal transduction, etc. (13). These cascades mainly include MAPKs (Slt2/Hog1/Fus3), MAPK kinases (MAPKK, Mkk1/Pbs2/Ste7), and MAPK-kinase kinases (MAPKKK, Bck1/Ste11/Ssk2). Once the MAPK pathway is activated, transcription factors are phosphorylated and transmitted to the nucleus, initiating targeted gene expression (14). Five MAPK pathways have been identified in Saccharomyces cerevisiae, which have been proven to be related to reproduction, cell wall integrity, invasive growth, hyperosmotic adaption, and conidiation (15). The Slt2, Hog1, and Fus3/Kss1 branches constitute the cell wall integrity (CWI) pathway, hyperosmotic glycerol (HOG) pathway, and filamentous (invasive hypha) growth pathway, respectively (15, 16). In Candida albicans, four MAPK pathways have been characterized (17, 18). In filamentous fungi, however, only three MAPK pathways have been identified (Slt2, Hog1, Fus3/Kss1), such as in Aspergillus nidulans, Magnaporthe oryzae, Fusarium graminearum, Neurospora crassa, and Metarhizium anisopliae (19–22).

Two-component systems (TCSs), which are widely present in bacteria, higher plants, and fungi, are involved in cell osmotic stress adaptation, cell cycle, growth and development, and secondary metabolites (23). The basic TCS pathway mainly involves two multidomain proteins: a histidine protein kinase (HPK) whose autokinase activity is dependent upon an environmental stimulus, and a response regulator (RR), onto which a phosphoryl group is transferred from the phosphorylated HPK; this mediates phosphorylation-dependent effects within the cell, thus regulating fungal adaption to multiple stresses (24–26).

As an upstream membrane protein of the SLN1 branch of the HOG-MAPK pathway, Sln1 plays various roles in different species. In S. cerevisiae, Sln1 is indispensable for fungal growth (27). In Aspergillus nidulans, deletion of *AnTcsB* (the homologue of *Sln1*) has no effect on mycelial development and morphology (28). In the human fungal pathogen Candida albicans, a *CaSln1*-deletion mutant showed decreased virulence, inhibited mycelial development, and slightly decreased tolerance to high osmotic stress (29). In Talaromyces (formerly *Penicillium*) marneffei, a *PmSlnA* (the homologue of *Sln1*)-deletion mutant exhibited delayed conidial germination and yield and increased susceptibility to high osmotic pressure and cell wall-disturbing chemicals (30). Moreover, the *PmSlnA* mutant showed an abnormal distribution of chitin during hypha branching and an inability to form yeast cells against hosts, thus decreasing its virulence (30). In the phytopathogenic fungus Colletotrichum lindemuthianum, a *ClSln1* mutant exhibited a loss of pathogenicity against the host plant and a less-melanized appressorium (31). In Botrytis cinerea, deletion of *BcSln1* changed the shape and morphogenesis of conidia but did not affect fungal virulence (32). In Fusarium graminearum, *FgSln1* deletion contributed nothing to conidial germination or conidial yield; however, the mutant showed a decreased ability to penetrate the host cells (19). In Magnaporthe oryzae, a *MaSln1* mutant presented altered tolerance to cell wall-disturbing chemicals and oxidative and osmotic stresses, as well as totally losing its ability to infect plant cells (33). In the entomopathogenic fungus Nomuraea rileyi, a *NrSln1*-knockdown mutant showed decreased conidial yield and reduced pathogenicity (34). In conclusion, Sln1 plays distinct roles in different fungi. In entomopathogenic fungi, however, the functions of Sln1 have not yet been clarified systematically.

In this research, we clarified the roles of *MaSln1* (the homologue of *Sln1*) in the model fungal pathogen Metarhizium acridum through gene knockout and rescue strategies. Our results showed that the deletion of *MaSln1* did not affect conidial germination, conidial yield, or resistance to most of the chemical agents tested. However, fungal sensitivity to UV-B and heat shock were significantly increased in Δ*MaSln1*. Bioassays showed that fungal pathogenicity was significantly reduced when *MaSln1* was deleted. Interestingly, because MaSln1 is an upstream transmembrane protein, its regulation of the conidiation pattern shift in SYA medium may be independent of the conserved MAPK pathway in *M. acridum*. RNA-seq was used to reveal the mechanism by which MaSln1 regulates the conidiation pattern shift. The results indicated that MaSln1 may regulate the expression of genes related to cell division, mycelial growth, and cytoskeleton remolding, thus regulating the conidiation pattern shift in *M. acridum*.

## RESULTS

### Identification of *MaSln1* and validation of mutant strains.

**FIG 1:**
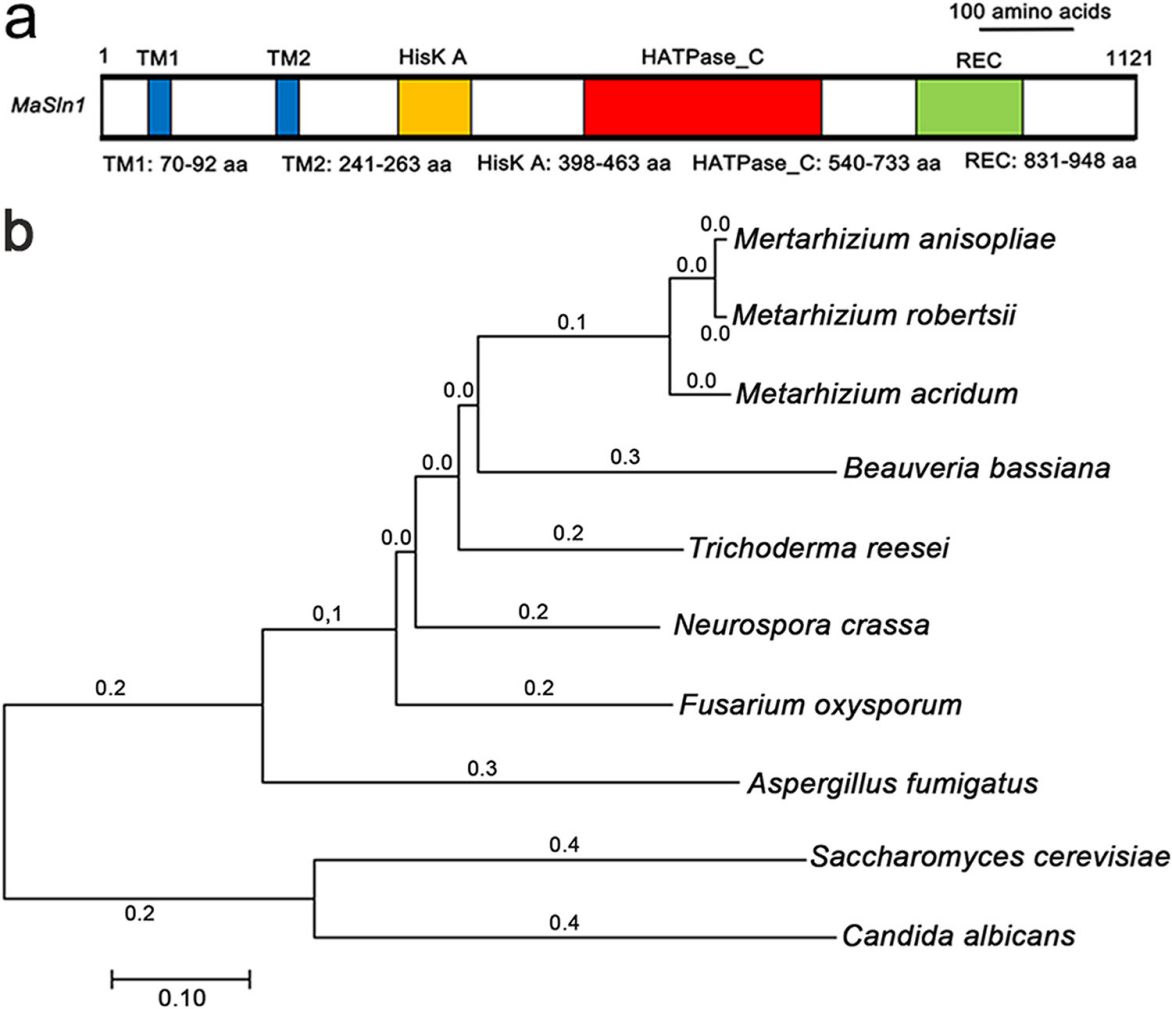
Characterization of *MaSln1* and construction of phylogenetic tree by bioinformatic analysis. (a) Schematic illustration of conserved domain in MaSln1. (b) Construction of a phylogenetic tree by using the Sln1 protein sequences of Metarhizium *acridum* (XP_007812240.1), Neurospora crassa (XP_958858.1), Beauveria bassiana (PMB73320.1), Metarhizium *anisopliae* (KAF5138022.1), Metarhizium *robertsii* (XP_007816648.1), Saccharomyces cerevisiae (CAA86131.1), Fusarium oxysporum (EWY90812.1), Trichoderma reesei (XP_006969916.1), Aspergillus fumigatus (XP_001481640.1), Candida albicans (KHC30928.1).

The full-length *MaSln1* (MAC_05900) coding sequence is 3,426 bp with a 60-bp intron, encoding a protein with 1,121 amino acids. The isoelectric point and molecular weight are 6.88 and 122.8 kDa, respectively, as determined through bioinformatic analysis. The web resource SMART (http://smart.embl.de/) was used to predict the conserved domain of *MaSln1*. Three transmembrane domains (TM), a histidine kinase domain (HiskA), a phosphor-transferase domain (HATPase_C), and a receptor domain (REC) are included (Fig. 1a). The neighbor-joining method was applied to construct a phylogenetic tree of MaSln1 (Fig. 1b) using MEGA v7.0, indicating that *MaSln1* is conserved in fungi based on sequence homology. The homologue recombination deletion and rescue strategies were adopted to obtain deletion and complementation vectors, respectively (Fig. S2a and b in the supplemental material). Southern blotting revealed that *MaSln1* was successfully deleted and rescued (Fig. S2c).

### Deletion of *MaSln1* had little or no effects on conidial germination or conidial yield.

To access the effect of *MaSln1* on fungal growth, conidial germination rates and conidial yields were both counted. Compared with that of the wild-type (WT) and complemented mutant (CP) strains, conidia germinated slowly on 1/4 Sabouraud dextrose agar plus yeast (SDAY) medium when incubated for 4 or 6 h (Fig. S3a; *P < *0.05), but reached the same level after 8 h incubation. The half-germination times (GT_50_s) of the strains showed no statistical differences (Fig. S3b; *P > *0.05). Additionally, deletion of *MaSln1* did not affect conidial yield (Fig. S3c).

### Deletion of *MaSln1* increased fungal sensitivity to heat shock and UV-B irradiation and increased fungal tolerance to oxidative stress.

**FIG 2:**
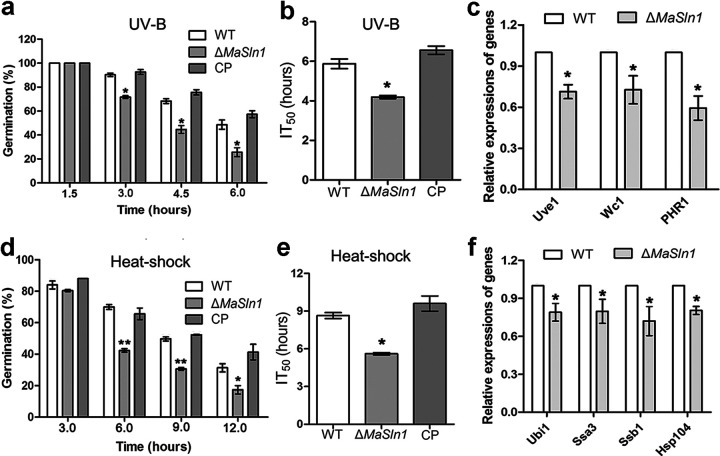
Stress tolerance assays for UV-B irradiation and heat shock in fungal strains. (a) Germination of fungal conidia treated with UV-B irradiation at 1,350 mW/m^2^ for 1.5, 3.0, 4.5, and 6.0 h. (b) Half-inhibition times of germination (IT_50_s) of fungal strains treated with UV-B. (c) Expression of genes involved in UV-B resistance in fungal strains. (d) Germination rates of fungal conidia treated with heat shock at 45°C for 3, 6, 9, and 12 h. (e) IT_50_s treated with heat shock. (f) Expression of genes involved in heat shock resistance in fungal strains. *, *P < *0.05; **, *P < *0.01. Error bars indicate standard deviations. WT, wild type; Δ*MaSln1*, *MaSln1*-deletion mutant; CP, complemented transformant.

To determine fungal sensitivity to UV-B irradiation and heat shock, conidial germination rates were calculated. UV-B tolerance in *MaSln1*-deletion mutants was significantly impaired (Fig. 2a). The half-inhibition times of germination (IT_50_s) by UV-B irradiation for the WT and CP strains were 5.87 ± 0.42 h and 6.56 ± 0.36 h, respectively, while the average IT_50_ of the *MaSln1*-deletion mutant was 4.19 ± 0.14 h (Fig. 2b; *P < *0.05). Reverse transcription-quantitative PCR (qRT-PCR) results revealed that the expression levels of genes related to DNA damage repair were all significantly decreased in the *MaSln1* mutant compared with those in the WT (Fig. 2c). Similarly, the sensitivity of the *MaSln1*-deletion mutant to heat shock was also increased (Fig. 2d; *P* < 0.01). The IT_50_ of Δ*MaSln1* was 5.60 ± 0.17 h, significantly decreased compared to that of the WT (8.65 ± 0.42 h) and CP (9.60 ± 1.04 h, Fig. 2e; *P < *0.05). Expressions of genes related to heat shock were also downregulated in Δ*MaSln1* (Fig. 2f). In addition, we found that a *MaSln1*-deletion mutant showed reduced sensitivity to the oxidative stressor menadione compared to the WT and CP strains. However, no significant differences were found among WT, Δ*MaSln1*, and CP strains when they were supplied with other chemical agents (Fig. S4).

### Deletion of *MaSln1* reduced fungal virulence only during topical inoculation.

**FIG 3:**
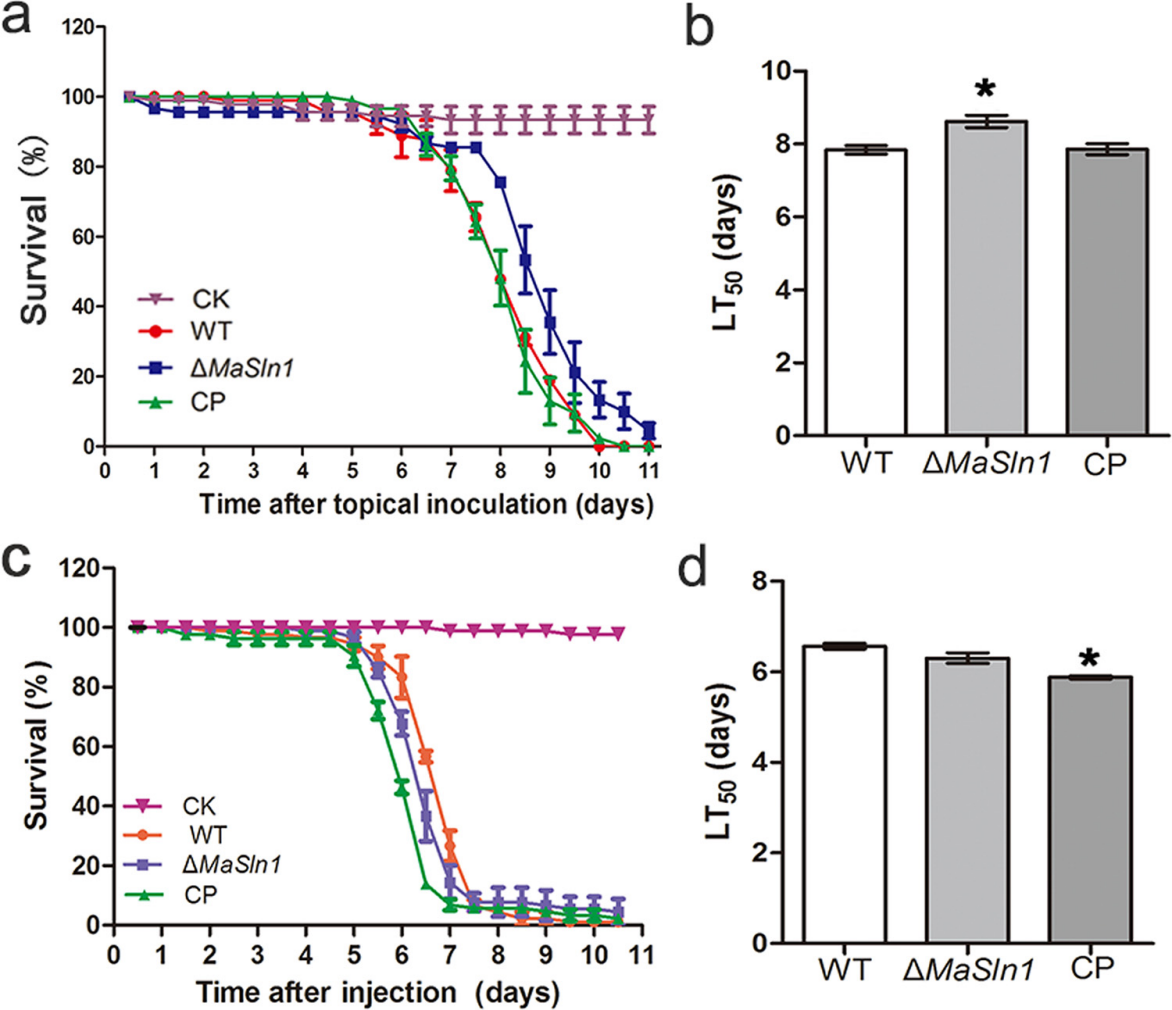
Virulence assays. (a) Survival of locusts inoculated with 5-μL conidia suspensions prepared in paraffin oil. Negative control was inoculated with 5 μL paraffin oil. (b) Half-lethal times (LT_50_s) of inoculation assays. (c) Survival of locusts injected with 5-μL conidia suspensions prepared in double-distilled water (ddH_2_O). Negative control was inoculated with 5 μL ddH_2_O. (d) LT_50_s of injection assays. *, *P < *0.05. Error bars indicate standard deviations.

Bioassays were performed using two methods. Topical inoculation showed that the survival of Δ*MaSln1* was significantly increased compared to that of the WT and CP, (Fig. 3a; *P < *0.05). The half-lethal times (LT_50_s) for the WT, Δ*MaSln1*, and CP were 7.84 ± 0.19, 8.62 ± 0.29, and 7.86 ± 0.26 days, respectively (Fig. 3b; *P < *0.05). However, the LT_50_s of these three strains when they were administered via intrahemocoel injection showed no significant differences (Fig. 3c and d; *P > *0.05). These results indicated that *MaSln1* contributes to fungal virulence during adhesion and/or cuticle penetration.

**FIG 4:**
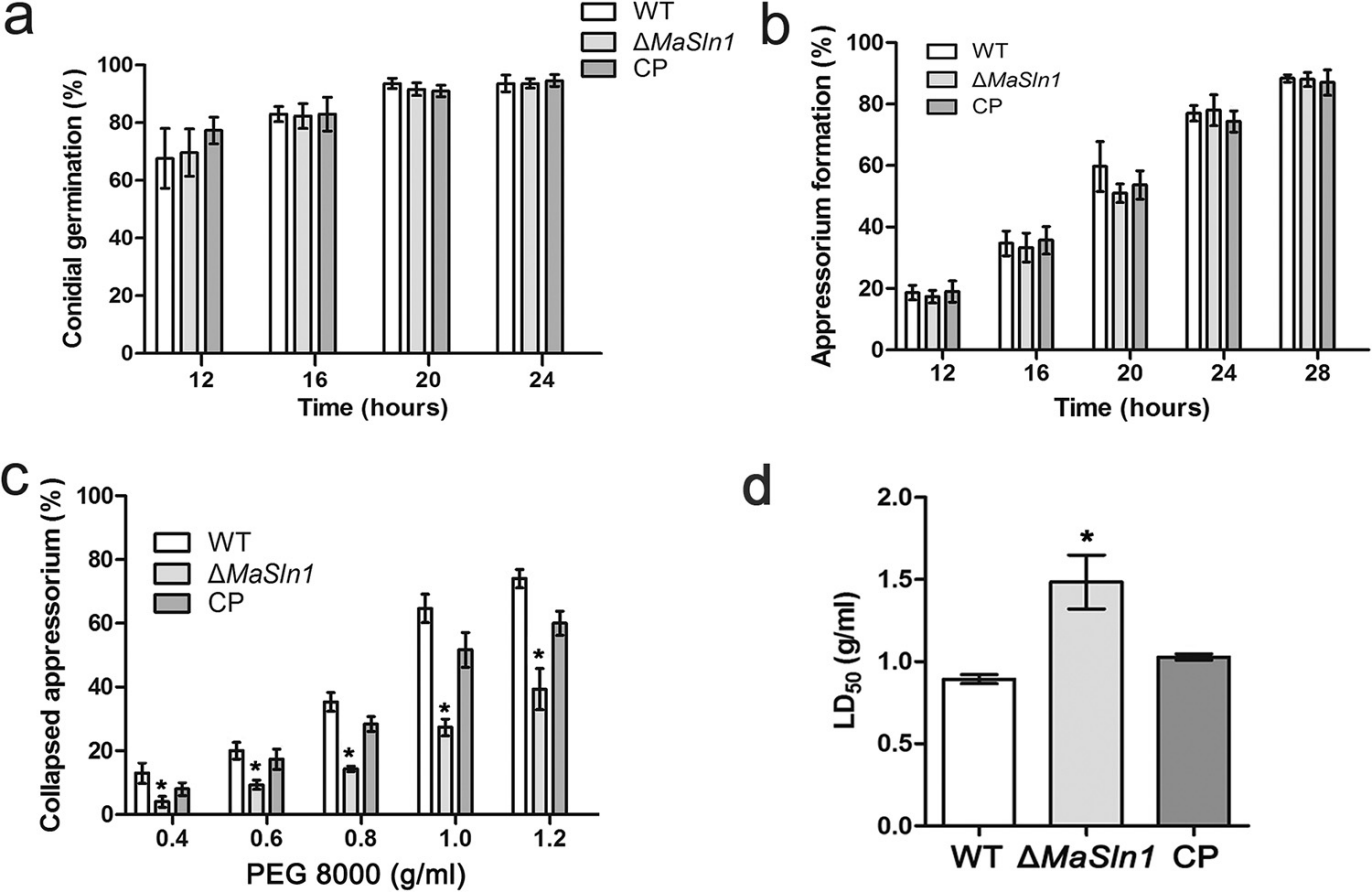
Effects of *MaSln1*-disruption on conidial germination, appressorium formation on locust wings, and turgor pressure. (a) Germination rates on locust wings. (b) Appressorium formation of different fungal conidia on locust wings. (c) Collapsed appressoria of fungal strains treated with different concentrations of PEG-8000. (d) Concentrations of PEG-8000 required to collapse 50% of appressoria. *, *P < *0.05. Error bars indicate standard deviations.

Further studies were conducted to explain the reason for the decreased virulence during topical inoculation. Unexpectedly, deletion of *MaSln1* contributed nothing to appressorium formation and conidial germination on locust wings (Fig. 4a and b; *P > *0.05). However, the rate of collapsed appressoria was significantly reduced in *MaSln1* mutants treated with PEG-8000 (Fig. 4c), indicating increased appressorial turgor pressure in Δ*MaSln1*. The LD_50_s of WT, Δ*MaSln1*, and CP strains were 0.89 ± 0.05 g/mL, 1.49 ± 0.28 g/mL, and 1.02 ± 0.34 g/mL, respectively (Fig. 4d; *P* *< *0.05).

### *MaSln1* shifting the conidiation pattern was independent of the conserved MAPK signaling pathway.

**FIG 5:**
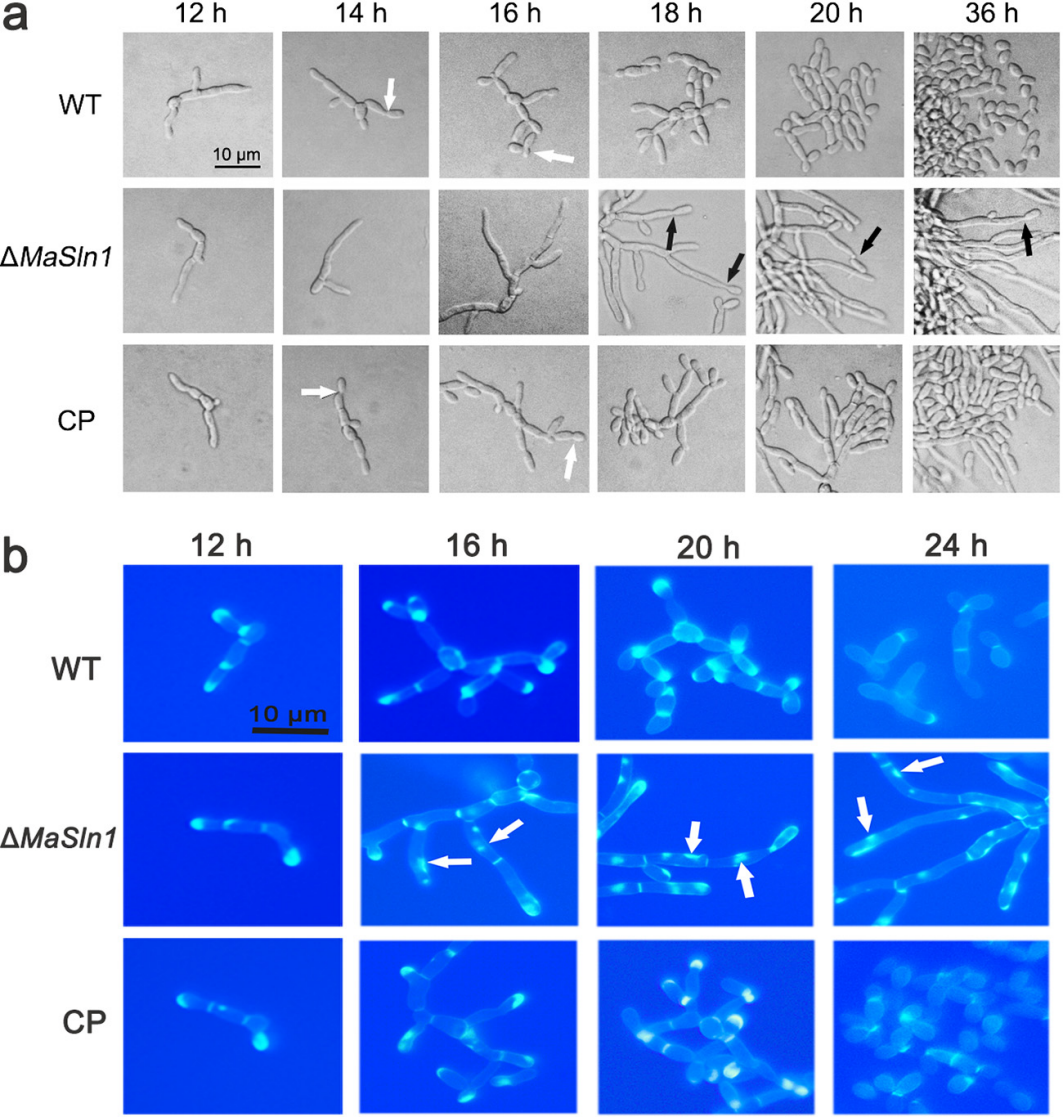
Effects of *MaSln1*-disruption on conidiation pattern. (a) Conidiation patterns of different fungal strains on SYA medium. Black arrows indicate normal conidiation. White arrows indicate microcycle conidiation. (b) Microscopic observation of chitin in hypha stained by Calcofluor white. White arrows indicate irregular distributions of chitin.

Compared with the WT and CP strains, the *MaSln1* deletion strain exhibited a different conidiation pattern on SYA medium. At 14 h, the WT and CP strains began to produce conidia with arrested hypha elongation (white arrows in Fig. 5a). At 16 h, they exhibited typical microcycle conidiation, while the *MaSln1* mutant continued growing with long hyphae. Until 18 h, the *MaSln1* mutant began to form conidiophores at the apex of hyphae (black arrow in Fig. 5a). At 36 h, WT and CP strains generated a lot of conidia through microcycle conidiation; the *MaSln1* mutant, however, still exhibited normal conidiation with long hyphae (Fig. 5a). These results illustrated that the absence of *MaSln1* could shift the conidiation pattern in *M. acridum*. Calcofluor white (CFW)-staining revealed that *MaSln1* contributed to chitin distribution in hyphae. Compared with that in the WT and CP strains, the chitin in Δ*MaSln1* was distributed not only in the septa and the tips of hyphae, but also irregularly in the extended hyphae (Fig. 5b).

**FIG 6:**
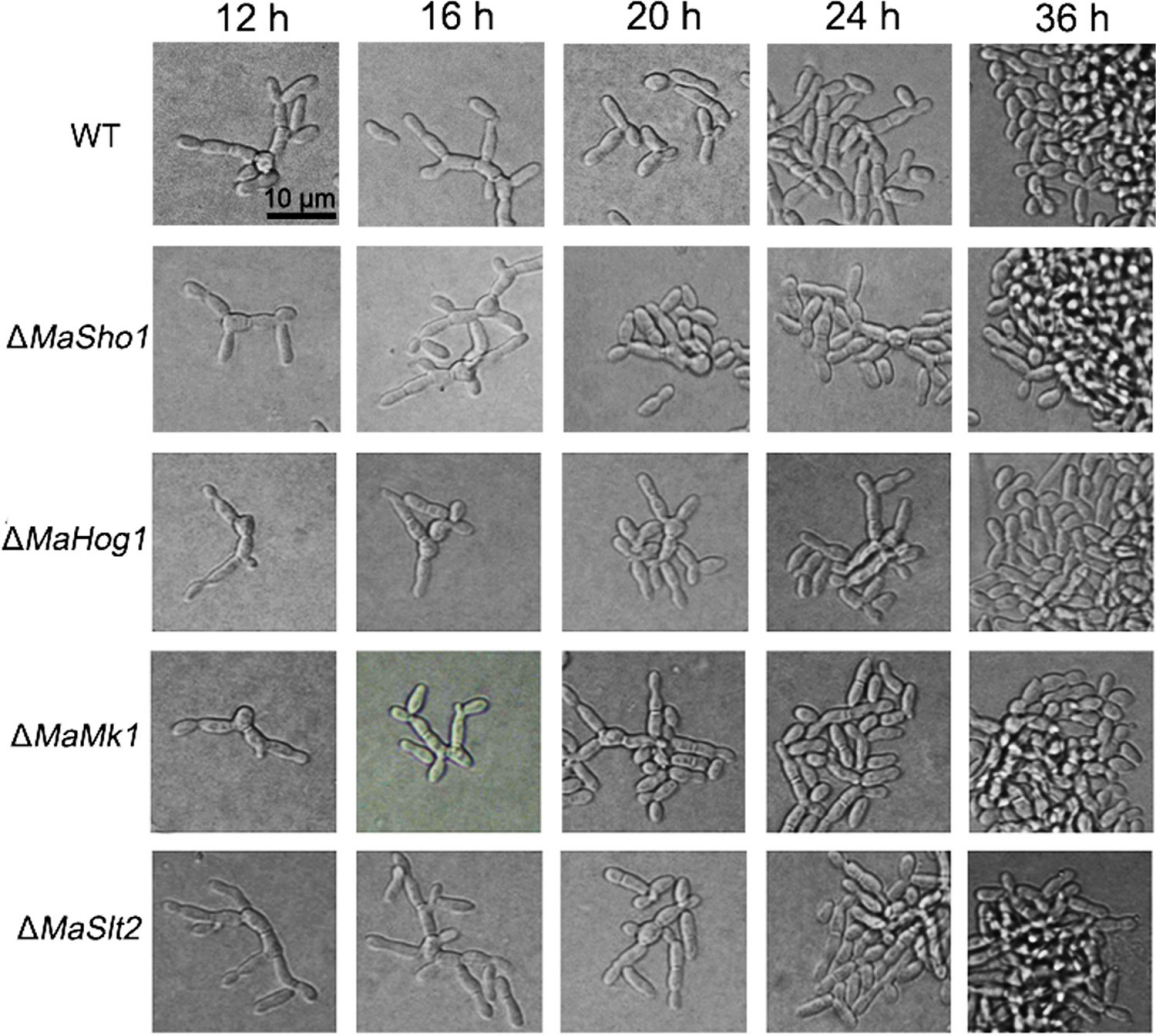
Conidiation pattern analyses of fungal strains related to the MAPK signaling pathway on SYA medium.

Because MaSln1 is an upstream sensor protein of the conserved HOG-MAPK signaling pathway, deletion of *MaSln1* shifted the conidiation pattern of *M. acridum*. To reveal the role of the MAPK pathway in conidiation pattern shifts in *M. acridum*, we observed the conidiation processes of some other mutants, Δ*MaSho1*, Δ*MaHog1*, Δ*MaSlt2*, and Δ*MaMk1*, involved in the MAPK pathway. It was found that the conidiation pattern was not changed when the transmembrane protein MaSho1 or the kinases MaHog1, MaMk1, or MaSlt2 were deleted (Fig. 6), and all the mutants exhibited the same microcycle conidiation pattern as the WT; this indicates a possibility that *MaSln1* contributes to shifting the conidiation pattern in *M. acridum*, independent of the conserved MAPK signaling pathway.

### Identification of DEGs during conidiation pattern shift.

**FIG 7:**
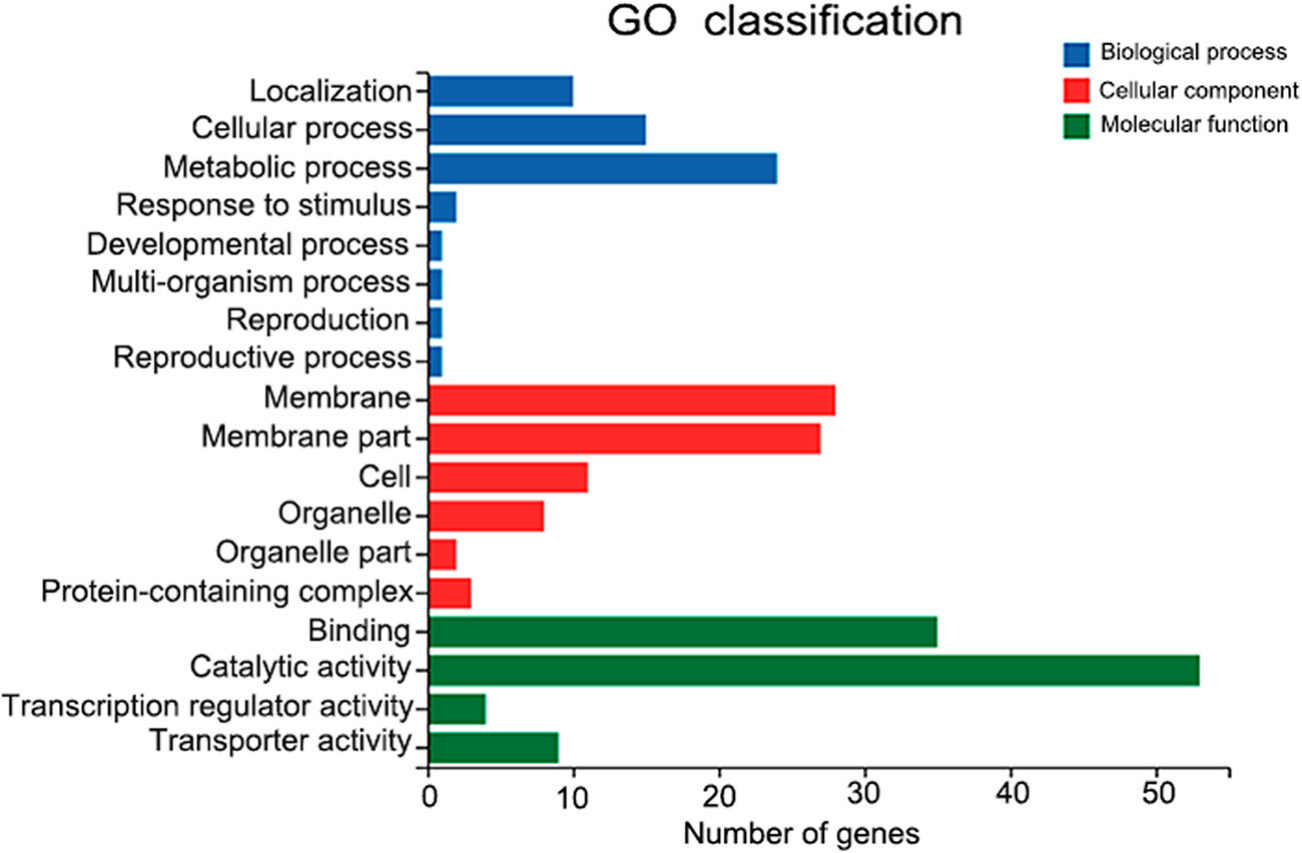
Gene ontology annotation of differentially expressed genes.

To reveal the role of *MaSln1* in conidiation pattern shifts, the genes regulated by MaSln1 during the conidiation pattern shift were identified by RNA-seq. According to the results of microscopic observation (Fig. 5a) and *MaSln1* expression during the conidiation pattern shift (Fig. S5), samples of WT and Δ*MaSln1* cultured on SYA medium for 14 h were collected for analysis. A total of 143 DEGs were isolated to be further analyzed. Among the 143 DEGs, 61 (∼42.7%) were upregulated and 82 (∼57.3%) were downregulated (Fig. 7a). Among these, 39 DEGs (∼27.3%) were annotated as hypothetical proteins (Table S2). To validate the reliability of the RNA-seq data, 21 DEGs were selected for qRT-PCR analysis. Results revealed that, except for three genes that showed opposite directions, 18 genes (∼85.7%) exhibited similar patterns to those in the RNA-seq data (Fig. S6), indicating that the RNA-seq data were reliable. Gene ontology (GO) analysis revealed that the 143 DEGs were enriched in 18 categories, and some genes related to metabolic processes, cellular processes, development processes, cell proliferation, and stimuli were differentially expressed in Δ*MaSln1* (Fig. 7b). Among these 98 known genes, no differentially expressed genes were found to be involved in the MAPK signaling pathway, confirming that *MaSln1*-regulated shifts in the conidiation pattern were probably independent of the MAPK pathway. Genes contributing to conidiation pattern shifts are presented in Table S3. One downregulated and three upregulated DEGs were related to fungal conidiation and cell growth, e.g., genes for a putative glyoxal oxidase precursor (MAC_08656), a surface protein (Mas1) (MAC_03649), a dual-specificity phosphatase Yvh1 (MAC_01700), and a pantothenate transporter (MAC_06827). In addition, genes related to cell cycle and regulation of cell differentiation were also dramatically up- or downregulated in Δ*MaSln1*. Three DEGs were involved in cell cycle, such as genes for an ATG5 protein (MAC_03903); a multidrug-resistance protein, Tpo1 (MAC_03963); and a putative oxidoreductase. Additionally, three genes involved in cytoskeleton remodeling were also remarkably downregulated. As a result, *MaSln1* deletion probably shifts the conidiation pattern by regulating the expression levels of genes which are crucial for conidiation, cell growth, cell cycle, and cytoskeleton remodeling.

## DISCUSSION

Sln1 is a transmembrane protein upstream from the HOG-MAPK signaling pathway. In this study, gene knockout and recovery methods were used to obtain deletion and complementation strains of *MaSln1* in *M. acridum* to characterize its function. Results revealed that *MaSln1* contributed nothing to conidial germination and conidial yield, but disruption of *MaSln1* significantly increased fungal sensitivity to UV-B irradiation and heat shock. Bioassays revealed that disruption of *MaSln1* decreased fungal virulence only by topical infection. Interestingly, because MaSln1 is an upstream sensor protein of the HOG-MAPK signaling pathway, deletion of *MaSln1* shifted the conidiation pattern on SYA medium. We also found that other genes (*MaSho1*, *MaHog1*, *MaSlt2*, and *MaMk1*) in the MAPK pathway were not involved in the conidiation pattern shift on SYA medium, indicating that *MaSln1* regulation of the conidiation pattern might not rely on the conserved MAPK pathway. Therefore, RNA-seq was used to screen the DEGs regulated by *MaSln1* during a conidiation pattern shift.

Conidial yield could affect the actual production efficiency of mycopesticides. Our study revealed that deletion of *MaSln1* does not affect conidial germination or conidial yield, consistent with observations in a *FgSln1*-deletion mutant of F. graminearum (19). However, different results were found in *T. marneffei* (30), *N. rileyi* (34), and *B. cinerea* (32), indicating that a conserved transmembrane protein played distinct roles in fungal growth and conidiation.

High temperature and UV irradiation can act as key factors affecting the efficiency of fungal insecticides in the field (35). In this study, deletion of *MaSln1* increased fungal sensitivity to heat shock and UV-B irradiation. Previous studies have shown that heat stimulation can affect conidial vitality and activate thermal protection-related mechanisms in *M. anisopliae* (36). Heat shock proteins (HSPs) play a key role in fungal heat stress (37, 38). Quantitative RT-PCR revealed that the expression of certain genes (*MaHsp101*, *MaSsa3*, *MaSsb1*, and *MaUbi1*) was significantly downregulated in Δ*MaSln1* compared with that in the WT, indicating that MaSln1 might contribute to thermotolerance in *M. acridum* by regulating the expression of genes related to heat shock response. In addition, genes involved in DNA repair (*MaUve1*, *MaWc1*, and *MaPHR1*) in Δ*MaSln1* were also downregulated, indicating that MaSln1 may also affect these genes to regulate fungal UV-B resistance. In M. oryzae, deletion of *Sln1* reduced fungal sensitivity to CFW and increased sensitivity to oxidative and osmotic pressure (33). In this study, however, the absence of *MaSln1* did not affect fungal tolerance to cell wall-disturbing chemicals. It was speculated that the other transmembrane proteins upstream from the HOG-MAPK pathway may make compensatory contributions to stress tolerance in *M. acridum*. Meanwhile, our results also indicated that the conserved sensor protein Sln1 displayed distinct functions in different fungi.

Virulence is also an important factor in evaluating the potential of biocontrol fungi. In this study, deletion of *MaSln1* impaired fungal virulence towards locusts, consist with the findings that a *NrSln1*-knockdown mutant of *N. rileyi* exhibited reduced virulence (34) and a *MaSln1*-deletion mutant of M. oryzae displayed a complete loss of virulence (33, 39). Further studies revealed that deletion of *MaSln1* had no effect on conidial germination or appressorium formation on locust hindwings, but it specifically increased appressorium pressure. Similar results were reported in M. oryzae, where deletion of *MoSln1* significantly increased appressorium turgor; however, the fungal virulence was totally lost (33, 39). Studies have shown that successful penetration mainly depends on the mechanical pressure of the appressorium (40). RNA-seq analysis revealed that the expression of the melanin synthesis-related genes, *Rsy1* and *Buf1*, was significantly upregulated in Δ*Mosln1*, which may be the reason for the increased appressorium turgor pressure in M. oryzae; however, deletion of *Mosln1* prevented septin-ring formation, impairing blast infections (39). Our results are consistent with those in Δ*Mosln1*. Additionally, we speculated that the deletion of *MaSln1* decreased fungal virulence due to the incorrect formation of septins in *M. acridum* appressoria, which thus decreased fungal virulence toward locusts. On the other hand, there may be some differences between insect pathogenic fungi and phytopathogenic fungi in successful host infection. Plant-pathogenic fungi must pass through each cell wall to infect the host, which is a big challenge to successful infection. Entomopathogenic fungus, however, can reside in the host hemocoel to utilize nutrients once they penetrate the host cuticle (1). This is the difference between insect fungal pathogens and phytopathogens in the infection of their hosts.

It is worth mentioning that MaSln1 is involved in the conidiation pattern shift, probably independent of the conserved MAPK signaling pathway in *M. acridum*. In this study, we found that the deletion of *MaSln1* changed microcycle conidiation into normal conidiation on SYA medium. However, the other genes (*MaSho1*, *MaHog1*, *MaMk1*, and *MaSlt2*) located in the MAPK signaling pathway contributed nothing to the conidiation pattern shift. What is the possible mechanism by which MaSln1 affects conidiation pattern shifts in *M. acridum*? RNA-seq was conducted to screen certain DEGs. RNA-seq data indicated that some of these DEGs were involved in fungal growth, conidiation, cell cycle, and cell division. Therefore, we speculate that MaSln1 affects the expression of these genes to shift the conidiation pattern.

In some other fungi, *Sln1* was involved in mycelial morphology development (30, 32, 41). Among the DEGs we studied, some are related to conidiation and cell growth. A gene for putative glyoxal oxidase precursor (MAC_08656) was upregulated in Δ*MaSln1*. In Ustilago maydis, glyoxal oxidase was crucial for mycelial growth (42). *Yvh1*, a dual-specific phosphatase gene related to the regulation of sporulation in S. cerevisiae (43), was significantly upregulated (MAC_01700). Moreover, many genes related to conidiation were significantly downregulated, such as a short-chain dehydrogenase gene (MAC_06605) and a pantothenate transporter gene (MAC_06827), both of which are involved in asexual spore formation (44, 45). Some genes involved in cell division were expressed differentially. *MAD1*, a gene for an adhesin protein (MAC_00987) which plays important roles in conidia formation and cell cycle in *M. anisopliae* (46), was upregulated in Δ*MaSln1*. In addition, many genes related to actin formation were also differentially expressed, such as a downregulated gene for a C2-structural protein (MAC_00302) which can act as a molecular bridge by inhibiting the nucleation activity of actin, thus promoting cell membrane flow (47). A gene encoding the long-chain reactive protein PIL-1 (MAC_03800), an important cytoskeleton component in fission yeast, was also significantly downregulated (48). The gene for NlpC-like cell wall protein (MAC_00504) is crucial for diaphragm production (49). In Candida albicans, an actin cytoskeleton regulatory protein gene, *SLA1*, associated with mycelial growth (50), was significantly downregulated (MAC_05587). The cell surface protein gene *Mas1* (MAC_03649), which is important for fungal morphogenesis, was upregulated (51). In addition, some other genes related to cell cycle and cell division which are crucial for fungal growth, such as the genes for autophagy-related protein ATG5 (MAC_03903), multidrug resistance protein Tpo1 (MAC_03963), and oxidoreductase (MAC_04042) were also differently expressed. In M. oryzae, *MaSln1* is crucial for septin and actin reorganization during appressorium formation (39), suggesting that *MaSln1* may also affect the expression of genes involved in actin and septin formation during the conidiation pattern shift in *M. acridum*; this needs to be further explored.

In summary, *MaSln1* contributes to fungal virulence and stress tolerance in *M. acridum*. Interestingly, as a transmembrane protein located upstream from the conserved HOG-MAPK signaling pathway, MaSln1 shifted the conidiation pattern independent of the MAPK pathway. RNA-seq data revealed that some DEGs related to cell morphogenesis, cell cycle, and cell division may contribute to the conidiation pattern shift.

## MATERIALS AND METHODS

### Strains.

The wild-type strain *M. acridum* CQMa102, used for gene disruption construction, was cultured normally (52). The *MaSho1*-disruption (Δ*MaSho1*) (52), *MaHog1*-disruption (Δ*MaHog1*) (20), and *MaMk1*-disruption (Δ*MaMk1*) mutants (21) have been previously constructed in our lab. For DNA manipulation, Escherichia coli DH5α (Bioground Biotech, Beijing, China) was used. Agrobacterium tumefaciens AGL-1 was used for fungal transformation (Weidi Biotechnology, Shanghai, China). *MaSln1* was deleted using the split-marker strategy. To construct the *MaSln1*-deletion vectors, the primer pairs Sln1-LF/Sln1-LR and Sln1-RF/Sln1-RR were used to amplify the sequences up- and downstream from *MaSln1*. The resulting fragments were inserted into pK2-SM-F and pK2-SM-R to yield the *MaSln1*-deletion vectors pK2-SM-MaSln1-F and pK2-SM-MaSln1-R, which were transformed into the WT to screen the *MaSln1*-deletion strains. For *MaSln1* complementation, the 5′-flanking region and *MaSln1* open reading frame (∼5.6 kb) were cloned into the plasmid pK2-Sur-GFP, which harbored a *Sur* cassette, to yield pK2-MaSln1-Sur-GFP vector. The resultant vector was integrated into Δ*MaSln1*. The complemented mutants were screened on Czapek-Dox medium containing 20 μg/mL chlorimuron ethyl (MilliporeSigma, Bellefonte, PA, USA). Fungal transformation mediated by A. tumefaciens and transformant validation were performed as described previously (53). For *MaSlt2* deletion, the *bar* cassette was inserted into the coding sequence of *MaSlt2* to destroy its function, as described previously (20). The *MaSlt2*-disruption mutants were screened by PCR (Fig. S1). All the primers used in this study are listed in Table S1 in the supplemental material.

### Effects of gene deletion on phenotypic changes.

For the conidial germination assay, conidial suspensions (1 × 10^7^ cells/mL, 100 μL) of microbial strains in Tween 80 (0.05%) were inoculated evenly on 1/4 SDAY (1% glucose, 0.5% yeast extract, 0.25% peptone and 1.8% agar, w/v) plates, then incubated at 28°C. Germination percentage was examined every 2 h. For conidial yield, 2 μL of suspensions (1 × 10^7^ cells/mL) were inoculated into 24-well plates containing 1/4 SDAY (1 mL). After incubation at 28°C for 3, 6, 9, 12, and 15 days, conidia in each well were collected and suspended with 0.05% Tween 80, respectively, and examined with a hemocytometer. To access fungal sensitivities to cell wall-disturbing agents, conidia suspensions (2 μL, 1 × 10^6^ cells/mL) were inoculated on 1/4 SDAY or 1/4 SDAY with Congo Red (CR, 500 μg/mL) and Calcofluor white (30 μg/mL). Colonies were recorded by taking photos after 5 days cultivation. Fungal heat shock and UV-B tolerance assays were both conducted as described previously (53). For heat shock tolerance assays, conidial suspensions (1 × 10^7^ conidia/mL) in sterile centrifuge tubes were treated in a water bath at 45°C for 3.0, 6.0, 9.0, and 12.0 h, respectively, and 100 μL of treated suspensions were spread evenly on SDAY plates, then cultured in darkness at 28°C for 20 h. For UV-B tolerance assays, 100 μL of conidial suspensions (1 × 10^7^ conidia/mL) were spread evenly on SDAY plates. The plates were immediately exposed to UV-B radiance at a dose of 1,350 mW/m^2^ for 1.5, 3.0, 4.5, and 6.0 h, respectively. The plates were immediately incubated in darkness at 28°C for 20 h. Conidial germinations were detected through microscopic observation. Experiments were based on biological triplicates.

### Conidial pathogenicity.

Two infection modes, cuticle infection and intrahemocoel injection, were used to detect fungal virulence toward the fifth-instar locusts. For cuticle infection, conidial suspensions in paraffin oil (1 × 10^7^ cells/mL, 5 μL) were inoculated on locusts. Alternatively, locusts were injected via the hemocoel with 5-μL suspensions prepared in double-distilled water (ddH_2_O; 1 × 10^6^ conidia/mL). Negative controls for both methods were inoculated with 5 μL of pure paraffin oil or 5 μL sterile ddH_2_O. Survival of locusts was calculated every 12 h. Each group included 30 insects and was based on three replicates.

### Assays for appressorium formation.

Assays of appressorium formation were performed as reported previously (54). Briefly, locust wings were prepared and sterilized by autoclaving after cleaning with ddH_2_O. Next, the wings were immersed in conidial suspensions with concentrations of 1 × 10^7^ cells/mL, and subsequently cultured at 28°C for 12 to 28 h to examine conidial germination and appressorium formation. For appressorium turgor pressure examination, collapsed appressoria treated with PEG-8000 were counted (46). Experiments were based on biological triplicates.

### Characterization of conidiation pattern shifts and identification of differently expressed genes.

Conidial suspensions (1 × 10^7^ conidia/mL) of WT, Δ*MaSln1*, and CP strains were spread evenly onto SYA (3% sucrose, 0.5% yeast extract, 0.3% NaNO3, 0.05% MgSO4, 0.05% KCl, 0.1% KH2PO4, 0.001% FeSO4, 0.001% MnSO4, and 2% agar, w/v) medium. Fungal growth was recorded every 2 h by microscopic observation. CFW staining (1 μg/mL) was used to detect fungal cell wall chitin distribution (55). Total RNA was extracted using an Ultrapure RNA Kit (CoWin Biosciences, Beijing, China) according to the manufacturer’s protocol. Total RNA was qualified and quantified using a Nano Drop and an Agilent 2100 Bioanalyzer (Thermo Fisher Scientific, MA, USA). For mRNA library construction, mRNA purified with oligonucleotide(dT)-attached magnetic beads was fragmented into small pieces, which were synthesized into double-stranded cDNA by reverse transcription. After quality control, the product was validated using an Agilent 2100 Bioanalyzer. The double-stranded PCR products were heated, denatured, and circularized to obtain the final library. The single-stranded circle DNA was formatted as the final library, which was amplified using phi29 to make a DNA nanoball (DNB) with more than 300 copies of one molecule each; DNBs were loaded into the patterned nanoarray, and 100 paired-end base reads were generated on a BGIseq500 platform (BGI Group, Wuhan, China). The sequencing data were filtered using SOAPnuke (v1.5.2) (56) for quality control to obtain clean reads, which were mapped to the reference genome of *Metrahizium acridum* (57) using HISAT2 (v2.0.4) (58). Ericscript (v0.5.5) (59) was used to fuse genes and rMATS (V3.2.5) (60) was used to fuse differential splicing genes (DSGs). Bowtie2 (v2.2.5) (61) was adopted to align the clean reads to the gene set, and RSEM (v1.2.12) (62) was applied to calculate gene expression levels. Essentially, DESeq2 (v1.4.5) (63) was used for differential expression analysis with a *Q* value of ≤0.05. To annotate DEGs, GO enrichment analysis (http://www.geneontology.org/) was performed with phyper (https://en.wikipedia.org/wiki/Hypergeometric_distribution) based on the hypergeometric test. Bonferroni was used for significance level correction (*Q* value of ≤0.05) with a rigorous threshold. qRT-PCR was used to validate the reliability of the RNA-seq data, which are presented in Table S2.

### Statistical analysis.

A one-way analysis of variance was applied to analyze the phenotypic estimate based on triplicate tests. Tukey’s honest significant difference (HSD) test was used to determine statistical differences among the fungal strains.

### Data availability.

The RNA-seq data in this study have been deposited in the NCBI BioProject database under the accession number PRJNA739483.
