# Supplementary material for: MaSln1, a Conserved Histidine Protein Kinase, Contributes to Conidiation Pattern Shift Independent of the MAPK Pathway in Metarhizium acridum
Source: Microbiol Spectr. 2022 Mar 28;10(2):e02051-21. doi: 10.1128/spectrum.02051-21 (PMC9045129; doi:10.1128/spectrum.02051-21)
Supplement: SUPPLEMENTAL FILE 1 — Supplemental material. Download SPECTRUM02051-21_Supp_1_seq11.pdf, PDF file, 0.5 MB [file spectrum02051-21_supp_1_seq11.pdf]

For Microbiology Spectrum

MaSln1, a conserved histidine protein kinase, contributes to conidiation pattern shift independent of the MAPK pathway in *Metarhizium acridum*

Zhiqiong Wen[1,2,3], Yuxian Xia[1,2,3*], Kai Jin[1,2,3*]

1 Genetic Engineering Research Center, School of Life Sciences, Chongqing University, Chongqing 401331, PR China;

2 Chongqing Engineering Research Center for Fungal Insecticide, Chongqing 401331, PR China

3 Key Laboratory of Gene Function and Regulation Technologies Under Chongqing Municipal Education Commission, Chongqing 401331, PR China

*Correspondence: yuxianxia@cqu.edu.cn; jinkai@cqu.edu.cn

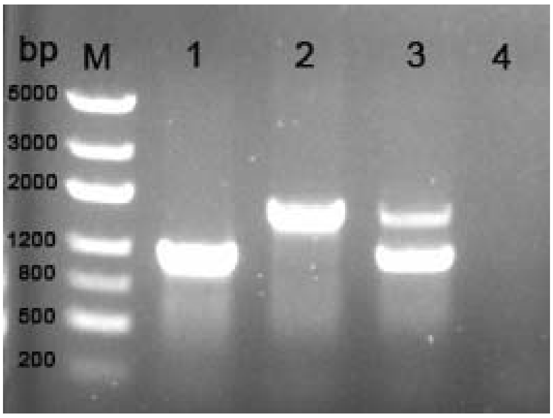

Fig. S1 Validation of Δ*MaSlt2* mutants by PCR analysis.

M: DNA marker. Lane 1, 2, 3, 4 represented WT, Δ*MaSlt2*-30#, Δ*MaSlt2*-87# and negative control, respectively. The Δ*MaSlt2*-30# were used for observation of conidiation pattern.

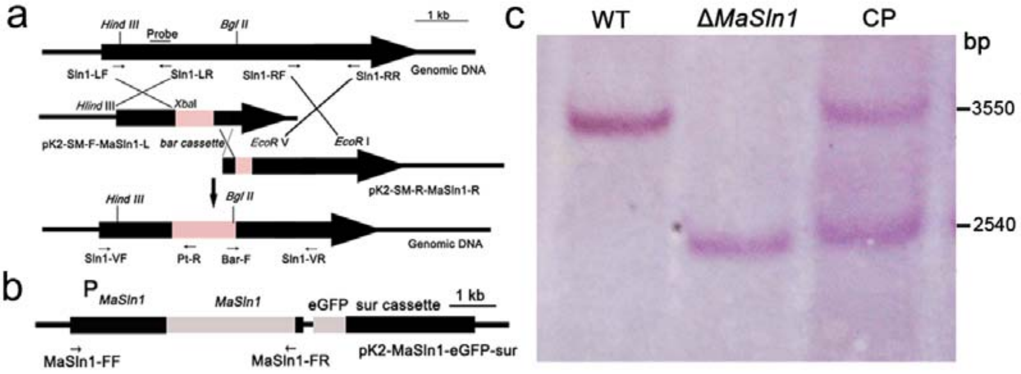

Fig. S2 Disruption and complementation of *MaSln1*.

(a) Schematic illustration of *MaSln1* disruption. (b) Design of the *MaSln1* complementation vector. (c) Southern blotting. Genomic DNA from WT, Δ*MaSln1* and CP were digested with *Bgl*II and *Hin*dIII. A 557 bp fragment of the *MaSln1* 5′ flanking sequence was used as the probe. WT: wild type; Δ*MaSln1*: the *MaSln1* disruption mutant; CP: complemented mutant.

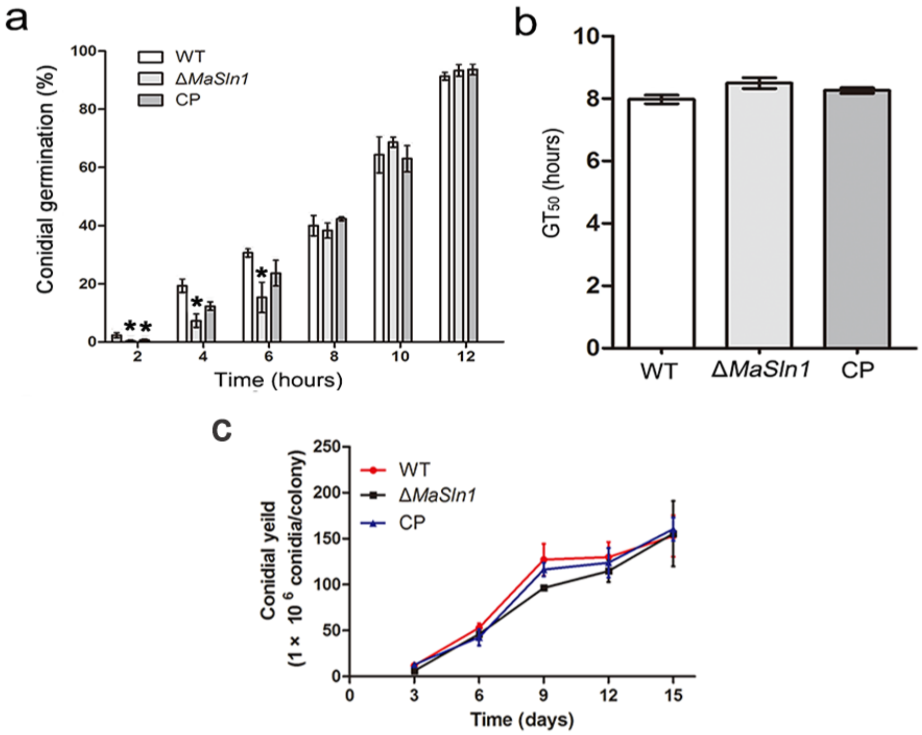

Fig. S3 Germination and conidiation assays of fungal strains. (a) Germination rates of fungal strains incubated for 2, 4, 6, 8, 10, 12 h on 1/4 SDAY medium. (b) GT_{50} s of fungal strains. (c) Conidial yields of fungal strains at 3, 6, 9, 12, 15 days on 1/4 SDAY medium. * indicates the significance at $P < 0.05$. Error bars indicate the standard deviations. WT: the wild type; Δ*MaSln1*: the *MaSln1* deletion mutant; CP: the complemented transformant.

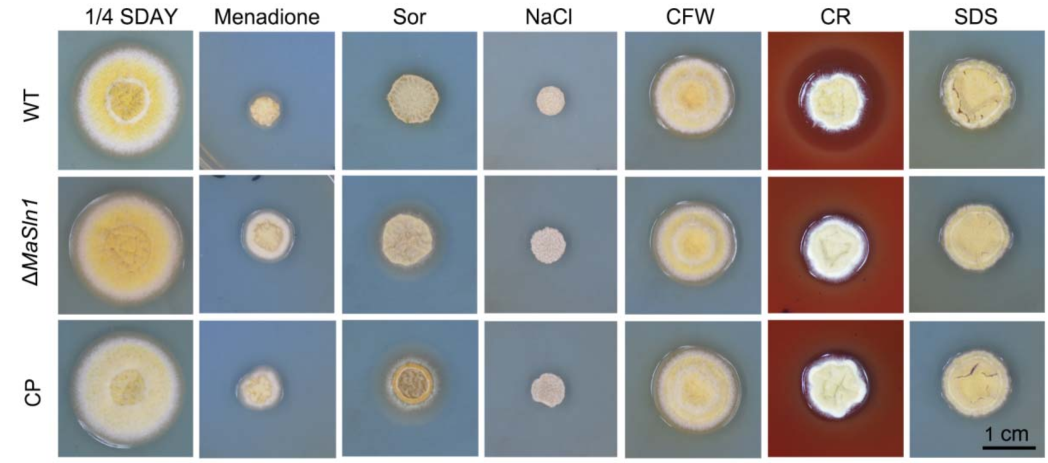

Fig. S4 Stress tolerance assays. Fungal colonies grown on 1/4 SDAY medium or 1/4 SDAY medium supplied with different chemical agents for 5 days.

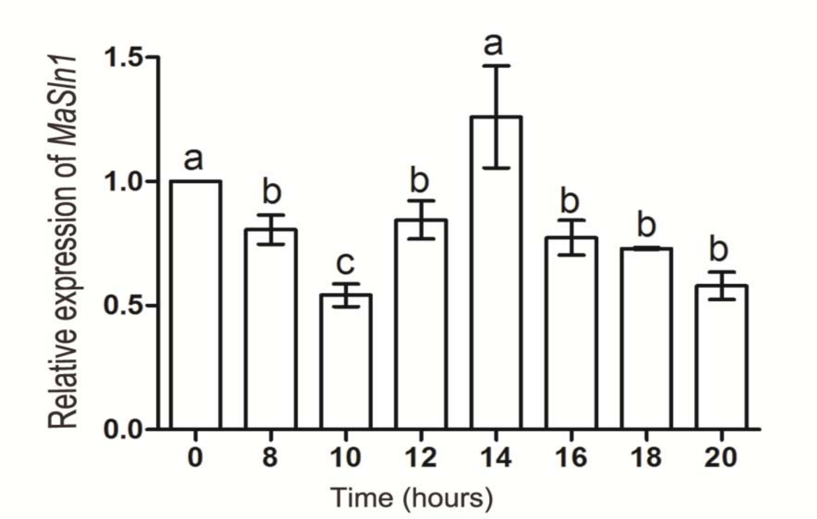

Fig. S5 Expression levels of *MaSln1* during conidiation in *M. acridum*.

a, *b* indicates significant difference at $P < 0.05$.

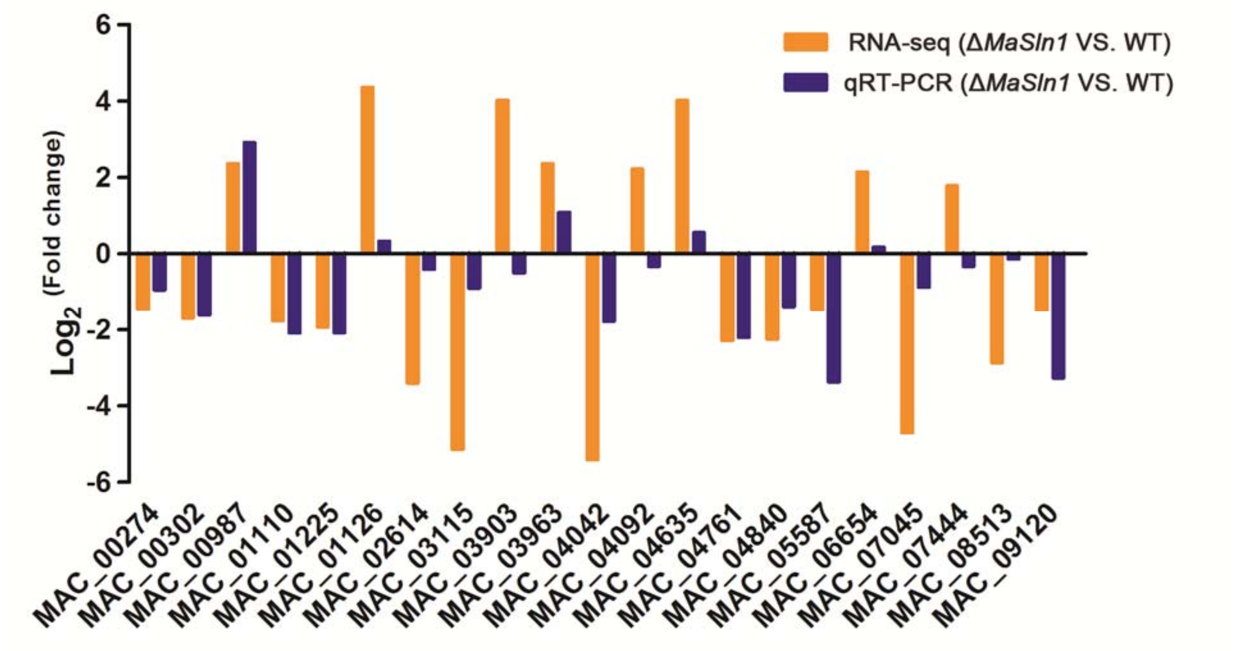

Fig. S6 Validation of the RNA-seq data by qRT-PCR.

Table S1 Primers used in this study.

Primers	Sequences (5′-3′)	Remarks
Sln1-LF	CAAAAGCCTCACAACGACCT	Used to construct the left arm of *MaSln1*-disruption vector
Sln1-LR	CATTATTCGGCGGCTTCAAC	
Sln1-RF	AGTAAGAGCCGATAAAGCG	Used to construct the right arm of *MaSln1*-disruption vector
Sln1-RR	GGCTACAAGAAGGGTGAA	
Bar-F	GCTCTACACCCACCTGCT	Used for screening the *MaSln1*-disruption mutants
Pt-R	CAGCCAAGCCCAAAAAGTG	
Sln1-VF	ATTAGCAAGGGGATTTAGAC	
Sln1-VR	TTCCTGTTACTACCTTGCTG	
Sln1-FF	ACTTTTTTGGTGGCTGGTG	Used to clone a ~1.9 kb fragment encompassing the *MaSln1* open reading frame and ~3.7 kb of 5' flanking sequence
Sln1-FR	CGCAGCCACTTCAGTCTCTT	
GFP-VR	CGATGCGGTTCACCAGGGTGT	Used for screening the *MaSn1* complemented mutants with Sln1-LF
Sln1-PF	AACACCTTCTTTCATCCCAT	Used to clone the probe
Sln1-PR	TTGTTGGCTTGATGGCTTGT	
Slt2-VF	TGAACCCATACGAACCTCCAT	Used to screen the *MaSlt2* disruption mutant
Slt2-VR	GCAGCTAGTAACATACTGG	
Sln1-qF	GAAGACGACGATGTTGTG	Used to analyze the expression of *MaSln1* by qRT-PCR
Sln1-qR	TCTAACTTGCTGGCTGAT	
Gpd-F	GACTGCCCGCATTGAGAAG	Primers of the endogenous control gene for qRT-PCR
Gpd-R	AGATGGAGGAGTTGGTGTTG	
HSP104-qF	CTGATTGAGAAGGAAGTC	Used to detect the gene expressions related to heat-shock
HSP104-qR	AACCGTAATCTTGTCATC	

**Primers**	**Sequences (5′-3′)**	**Remarks**
SSA3-qF	ATTCTGCTGCTTGATGTT	Used to detect the gene expressions related to heat-shock
SSA3-qR	TTCTTGGTGGGAATA GTG	
Ssb1-qF	CTCTCGGTGTCGCTATGG	
Ssb1-qR	TCGGCAACAGTGGTGAAG	
Ubi1-qF	CAAGAAGAAGGTCTACACTACTC	
Ubi1-qR	AATGTTGCCGTCCTTGTC	
PHR-qF	GAACAGACACTTGGAATC	Used to detect the expression of genes related to DNA damage repair
PHR-qR	TGCCTAAGTAGAGATATGC	
WC1-qF	TGTATCAACACTCCTTCAG	
WC1-qR	CGAGGTCAATAGCATCATA	
Uve1-qF	TAACAACGCACGAGATAT	
Uve1-qR	AGGCTATAACCATACTCTG	
MAC_00274-qF	GACCTCAAAGAATGGAAC	Used to validate the data of DGE by qRT-PCR
MAC_00274-qR	CGAAATAACCATGTCCAC	
MAC_00302-qF	GTAGTCATTGTTGGTATTG	
MAC_00987-qF	CTCCTGAGCAAGAGAATG	
MAC_00987-qR	AAGTTGAAGCCAGCATAG	
MAC_01110-qF	CTCGTCACCTTTCTTCAC	
MAC_01110-qR	CCTACTACACTGTCAATCTG	
MAC_01126-qF	CAGCAGCAGTTCAGAATC	
MAC_01126-qR	CCACGACCTCATCATCAT	
MAC_01225-qF	GTCCGCCATTGTCTCTAT	
MAC_01225-qR	ACGACTCCAAGATTGAAGA	
MAC_02614-qF	CAGACAATGGATGAACTTC	

**Primers**	**Sequences (5′-3′)**	**Remarks**
MAC_02614-qR	TGACTTCGCCAATATGAT	Used to validate the data of DGE by qRT-PCR
MAC_03115-qF	CATACGCTACATACTCCTTAC	
MAC_03115-qR	GATGTGCCGATACTTCAG	
MAC_03903-qF	TACCTCGCACTCCTACTC	
MAC_03903-qR	TCCTTGACACAGTTCAGAA	
MAC_03963-qF	GTATCCGCAGTTGTCTTG	
MAC_03963-qR	TACGAATCCACGAGGTAA	
MAC_04042-qF	TGGCAGGTTAGAGGTAATG	
MAC_04042-qR	GGCTGGATCTTGGTAATTG	
MAC_04092-qF	TCAGCGACGATACTTCTTC	
MAC_04092-qR	CTTAGAGCCGAACCAGAG	
MAC_04635-qF	GCCACTTCAAGATTGTCAA	
MAC_04635-qR	TTAGCCACAGCCATAACT	
MAC_04761-qF	GAACGCTGCTATTTCTGG	
MAC_04761-qR	TGTGGTAAAGTCAATTTCAAG	
MAC_04840-qF	ACACATATCCTCATATAACGAT	
MAC_04840-qR	TTACATTCCGCTGATCTC	
MAC_05587-qF	GCTATGCTCTTGCTTATACG	
MAC_05587-qR	TCCACGAATGCGATACTT	
MAC_06654-qF	TACTGTCCGATTGCTTCA	
MAC_06654-qR	CGAATGTAGAATGGTGAGAAT	
MAC_07045-qF	TCCGAATTGACCGTAACA	
MAC_07045-qR	GTAGCAAGTGAGCCATAAG	
MAC_07444-qF	ATTGTCTACTGATGTTGTC	

**Primers**	**Sequences (5′-3′)**	**Remarks**
MAC_07444-qR	TATGAACGATAGGCTTGA	Used to validate the data of DGE by qRT-PCR
MAC_08513-qF	GAGATGCTGGGCTTTTAC	
MAC_08513-qR	CTAACCATCACCAACCTC	

Table S2 Description of DEGs.

Gene ID	$\log_2(\Delta MaSln1/WT)$	P value (WT/Disruption)	Up or down regulation	Description
MAC_00141	-1.779605165	3.44E-06	Down	Hypothetical protein
MAC_00142	-1.701689354	3.07E-05	Down	Hypothetical protein
MAC_00168	-1.019255428	0.00631173	Down	n-alkane-inducible cytochrome P450
MAC_00171	1.414208667	6.61E-11	Up	Acetylcholinesterase precursor
MAC_00217	-1.035195961	1.63E-06	Down	Acyl-CoA dehydrogenase
MAC_00274	-1.44315346	0.000277675	Down	Enoyl-CoA hydratase/carnithine racemase
MAC_00301	-1.531895066	5.32E-12	Down	C2 domain protein
MAC_00302	-1.685197174	5.26E-19	Down	C2 domain protein
MAC_00504	-1.271047371	0.000945291	Down	NlpC/P60-like cell-wall peptidase, putative
MAC_00520	2.082546599	3.25E-07	Up	Hypothetical protein
MAC_00525	-1.174933345	3.90E-06	Down	Hypothetical protein
MAC_00584	1.146738012	9.24E-06	Up	Hypothetical protein
MAC_00611	1.327026469	5.48E-33	Up	Hypothetical protein
MAC_00655	-1.291868139	0.000805184	Down	C6 zinc finger domain-containing protein
MAC_00830	1.135386818	0.000192932	Up	Hypothetical protein
MAC_00948	1.122537405	0.000418723	Up	Hypothetical protein
MAC_00987	2.360359741	1.36E-55	Up	Adhesin protein Mad1
MAC_01097	-1.419128847	2.73E-44	Down	Mismatched base pair and cruciform DNA recognition protein, putative
MAC_01110	-1.747150329	1.06E-07	Down	Cytochrome P450, putative
MAC_01126	4.356925081	4.18E-29	Up	Hypothetical protein
MAC_01128	-1.101075603	0.005929702	Down	Hypothetical protein
MAC_01137	-1.305342479	4.35E-11	Down	Hypothetical protein
MAC_01225	-1.923631601	3.00E-17	Down	Glucose transporter-like protein

Gene ID	log2(Δ*MaSln1*/WT)	P value (WT/Disruption)	Up or down regulation	Description
MAC_01227	-1.087647062	0.000254276	Down	Periplasmic nitrate reductase, putative
MAC_01364	-1.024269806	0.000135808	Down	Hypothetical protein
MAC_01403	-1.206881786	0.000370615	Down	Hexose transport-related protein
MAC_01470	-1.728218692	1.31E-73	Down	Acyl-CoA dehydrogenase
MAC_01483	1.133304435	0.005563629	Up	Hypothetical protein
MAC_01700	1.171388214	0.004264303	Up	Dual specificity phosphatase, Yvh1
MAC_01725	2.213080302	7.48E-08	Up	DNA kinase/phosphatase Pnk1
MAC_01894	-1.140839103	8.36E-37	Down	Flavin-containing amine oxidasedehydrogenase, putative
MAC_01994	-1.126936587	0.005329769	Down	Hypothetical protein
MAC_02196	-1.20826577	0.0001105	Down	NmrA family transcriptional regulator
MAC_02353	1.041244412	2.11E-05	Up	Sodium/phosphate symporter, putative
MAC_02364	-1.009131736	0.001756894	Down	Hsp40 co-chaperone Jid1, putative
MAC_02614	-3.389688945	1.40E-17	Down	ESCRT-III component
MAC_02772	-1.241695662	9.42E-07	Down	DNA repair protein rad14
MAC_02980	1.833282717	8.34E-06	Up	Translation initiation protein sua5
MAC_03039	-1.097549546	4.21E-11	Down	Hypothetical protein
MAC_03052	1.134684047	1.63E-05	Up	Oxidoreductase, putative
MAC_03080	-1.068965873	2.41E-08	Down	Peroxisomal membrane protein (pmpp24), putative
MAC_03115	-5.132287453	1.23E-47	Down	Long chain fatty alcohol oxidase
MAC_03373	-4.633814654	2.06E-36	Down	L-asparaginase precursor
MAC_03481	-1.040361258	2.79E-06	Down	Electron transfer flavoprotein
MAC_03511	1.444192986	7.36E-06	Up	Exo-beta-1,3-glucanase
MAC_03561	-1.409430705	4.44E-08	Down	Nonspecific lipid-transfer protein precursor
MAC_03649	1.147475372	0.005285582	Up	Cell surface protein (Mas1)
MAC_03650	1.15678109	0.003543593	Up	Hypothetical protein
MAC_03800	-1.161280448	8.00E-11	Down	Sphingolipid long chain base-responsive Protein pil1

**Gene ID**	**log$_2$(Δ*MaSln1*/WT)**	**P value (WT/Disruption)**	**Up or down regulation**	**Description**
MAC_03845	-1.13479243	0.003344163	Down	Hypothetical protein
MAC_03903	4.029426049	9.49E-26	Up	ATG5 protein
MAC_03904	-1.384544881	9.73E-43	Down	ATG5 protein
MAC_03963	2.362507224	3.26E-13	Up	Multidrug resistant protein Tpo1
MAC_04042	-5.395728244	1.50E-54	Down	Oxidoreductase, 2-nitropropane dioxygenase family, putative
MAC_04092	2.217937012	6.15E-08	Up	Xaa-Pro dipeptidase
MAC_04229	1.173462914	0.000996491	Up	Endopolyphosphatase, ppn1
MAC_04279	1.168932113	5.11E-12	Up	Putative methionine permease
MAC_04378	1.064501383	6.22E-11	Up	Magnesium-translocating P-type Atpase family protein
MAC_04408	1.143565756	4.54E-11	Up	ABC transporter, ABCC1
MAC_04473	-1.009518642	0.000238533	Down	AIF-like mitochondrial oxidoreductase (Nfrl)
MAC_04477	-1.129820675	0.005779987	Down	Acetyltransferase
MAC_04517	1.377787414	0.000507451	Up	Bzip transcription factor
MAC_04635	4.017525857	5.31E-24	Up	Glutathione-dependent formaldehyde-activating gfa
MAC_04659	-1.118523071	1.88E-23	Down	Phosphoketolase
MAC_04708	1.11057161	7.80E-13	Up	Metacaspase casa
MAC_04761	-2.27901778	2.89E-20	Down	Hypothetical protein
MAC_04798	1.256768049	5.58E-08	Up	Hypothetical protein
MAC_04840	-2.240749401	1.99E-29	Down	Hypothetical protein
MAC_04881	-1.197957289	5.67E-61	Down	Pirin domain protein, putative
MAC_04965	-1.088453622	0.001315532	Down	Hypothetical protein
MAC_05022	-1.099649934	1.02E-11	Down	Hypothetical protein
MAC_05050	1.483401319	4.42E-08	Up	Brix domain containing protein, RPF1
MAC_05071	1.403769065	1.45E-08	Up	Hypothetical protein
MAC_05134	-1.310273183	2.40E-08	Down	Hypothetical protein

Gene ID	$\log_2(\Delta$*MaSln1*/WT)	P value (WT/Disruption)	Up or down regulation	Description
MAC_05169	-1.832225287	8.35E-06	Down	Hemerythrin HHE cation binding domain-containing protein
MAC_05428	-1.001296633	3.38E-05	Down	Hypothetical protein
MAC_05449	1.036495442	0.001743143	Up	Epoxide hydrolase 1
MAC_05455	-1.216463455	0.000597112	Down	Catechol dioxygenase, putative
MAC_05456	-1.355700283	4.83E-05	Down	Maleylacetate reductase
MAC_05500	-4.697359837	8.67E-40	Down	DUF500 domain protein
MAC_05587	-1.46340293	3.28E-13	Down	Actin cytoskeleton-regulatory complex protein sla1
MAC_05588	2.795751892	8.17E-12	Up	5'-nucleotidase precursor
MAC_05663	1.093068949	0.003276628	Up	RNA polymerase III subunit RPC82
MAC_05709	1.22549687	9.60E-13	Up	Uracil permease, putative
MAC_05814	-1.037526429	2.24E-05	Down	3-hydroxyacyl-coadehydrogenase
MAC_05843	-1.047667189	2.92E-15	Down	Maltase MLT3
MAC_05844	-1.173954071	5.82E-11	Down	MRT; a raffinose family of oligosaccharides transporter
MAC_05847	1.483198698	0.000198881	Up	V8-like Glu-specific endopeptidase
MAC_05900	-5.950660203	2.91E-69	Down	Histidine kinase Sln1
MAC_05908	1.717819753	5.95E-07	Up	Hypothetical protein
MAC_06123	-1.04571679	0.002989171	Down	Hypothetical protein
MAC_06190	1.641231333	6.03E-05	Up	Neutral trehalase
MAC_06244	1.477199087	0.000304718	Up	Methyltransferase
MAC_06270	-1.051386611	0.002963339	Down	Hypothetical protein
MAC_06283	-1.081370652	4.73E-83	Down	Acid trehalase
MAC_06329	1.284496093	2.97E-05	Up	Hypothetical protein
MAC_06548	3.487473999	3.49E-18	Up	Activator 138 kda subunit
MAC_06605	-1.17419326	1.34E-07	Down	Short-chain dehydrogenases/Reductase, putative
MAC_06610	1.147453806	0.005252933	Up	Beta-1,3-endoglucanase
MAC_06612	1.002507601	4.52E-07	Up	Cel5b putative endoglucanase

Gene ID	log$_2$(Δ*MaSln1*/WT)	P value (WT/Disruption)	Up or down regulation	Description
MAC_06654	2.149820627	1.64E-07	Up	Acriflavine sensitivity control Protein acr-2
MAC_06743	-1.276595889	1.05E-06	Down	Gephyrin
MAC_06750	1.069487657	0.003735401	Up	Hypothetical protein
MAC_06811	-1.095017745	0.00761965	Down	Alkaline serine protease AorO, putative
MAC_06827	-1.015818614	5.09E-05	Down	Pantothenate transporter
MAC_06829	-1.457290111	2.89E-08	Down	Acetamidase
MAC_07043	1.375653157	9.84E-10	Up	Glycerol-3-phosphate dehydrogenase
MAC_07045	-4.697891726	2.70E-37	Down	Glycerol kinase
MAC_07363	-1.55835943	1.65E-10	Down	Amidohydrolase family protein
MAC_07444	1.788478317	1.09E-05	Up	C6 transcription factor, putative
MAC_07810	1.041619696	2.54E-08	Up	DUF1212 domain membrane protein Prm10
MAC_07882	1.096111603	0.005912579	Up	Hypothetical protein
MAC_07930	-1.295461329	0.001335005	Down	Sterol esterase precursor
MAC_07958	1.482336618	0.000125147	Up	Tyrosinase, putative
MAC_07959	1.108896974	2.49E-05	Up	Hydantoinase/oxoprolinase, putative
MAC_08088	-1.477357447	1.04E-28	Down	Hypothetical protein
MAC_08103	-1.134824722	5.79E-09	Down	Flavoprotein family
MAC_08196	1.022427589	0.000186396	Up	Hypothetical protein
MAC_08222	-1.190832627	0.000700885	Down	Hypothetical protein
MAC_08299	1.375498509	0.000167891	Up	Hypothetical protein
MAC_08454	-1.461641068	4.91E-06	Down	Hypothetical protein
MAC_08513	-2.862277041	2.66E-12	Down	Alcohol dehydrogenase, Zinc-containing, putative
MAC_08656	1.119205627	2.49E-20	Up	Putative glyoxal oxidase precursor
MAC_08697	-1.090511803	0.002851742	Down	Hypothetical protein
MAC_08777	1.095782807	9.40E-05	Up	56kDa selenium binding protein (SBP56)
MAC_09120	-1.478923299	1.06E-13	Down	Putative neurofibromin, NF1

Gene ID	log$_2$(Δ*MaSln1*/WT)	P value (WT/Disruption)	Up or down regulation	Description
MAC_09145	-1.414607676	0.000343917	Down	Carboxylesterase family protein
MAC_09281	-1.009204195	2.52E-17	Down	C-4 methylsterol oxidase
MAC_09301	1.054051566	7.61E-05	Up	Putative protein arginine N-methyltransferase 3
MAC_09316	-1.094457083	0.000321109	Down	Aldo-keto reductase (AKR13)
MAC_09362	1.360587095	0.000580827	Up	Phosphate-repressible phosphate permease
MAC_09447	-1.162158927	0.004087677	Down	CRAL/TRIO domain protein
MAC_09504	-1.032807027	5.31E-05	Down	Putative glucoamylase GMY2
MAC_09508	-1.02262108	0.0009878	Down	Hypothetical protein
MAC_09617	-1.078858864	6.49E-70	Down	N-methyltransferase, putative
MAC_09811	-1.379269164	1.46E-06	Down	*O*-methyltransferase, putative
MAC_09945	-2.163551852	1.22E-07	Down	tRNA
MAC_10113	1.207011897	0.001227111	Up	Copper amine oxidase
MAC_10230	-1.209154667	0.003266901	Down	Xylitol dehydrogenase
MAC_10562	1.27491484	0.001440723	Up	Hypothetical protein
MAC_10565	1.176973456	0.000269955	Up	Hypothetical protein
MAC_10883	1.320842198	0.000330946	Up	Hypothetical protein

Table S3 Differentially expressed genes involved in conidiation pattern shift.

Gene ID	$Log_2^{(\Delta MaSln1/WT)}$	Description	Functions	References
MAC_08656	1.119205627	Putative glyoxal oxidase precursor	Contributed to virulence and mycelial growth	(1)
MAC_01700	1.171388214	Dual specificity phosphatase, Yvh1	Involved in conidiation	(2)
MAC_06605	-1.17419326	short-chain Dehydrogenases/reductase, putative	Contribute to fungal growth and conidiation	(3)
MAC_06827	-1.01581861	Pantothenate transporter	Contribute to life-span and conidiation	(4)
MAC_09120	-1.4789233	Putative neurofibromin， NF1	Involved in Ras signaling pathway which was related to mophogenesis and insexial development	(5)
MAC_00987	2.360359741	Adhesin protein Mad1	Required to orientate the cytoskeleton and stimulate the expression of genes involved in the cell cycle	(6)
MAC_00302	-1.68519717	C2 domain protein	Acted like a molecular bridge which promotes membrane deformation via its actin nucleation activity	(7)
MAC_03800	-1.16128045	Sphingolipid long chain Base-responsive protein PIL1	A novel component of the yeast cytoskeleton that implicated for the role of filament assembly in the spatial organization of cells	(8)
MAC_05587	-1.46340293	actin Cytoskeleton-regulatory complex protein SLA1	Involved in hyphal growth, actin patch dynamics and cell endocytosis	(9)
MAC_03649	1.147475372	Cell surface protein (Mas1)	Contributed to mitochondrial morphology and function	(10)
MAC_03903	4.029426049	ATG5 protein	Important for cell cycle G2/M arrest and involved in autophagy	(11)
MAC_03963	2.362507224	Multidrug resistant protein Tpo1	Contributed to cell cycle delay under oxidative stress	(12)
MAC_04042	-5.39572824	Oxidoreductase, 2-nitropropane dioxygenase family, putative	Involved in cell cycle	(13)

References

(1) Leuthner B, Aichinger C, Oehmen E, Koopmann E, Müller O, Müller P, Kahmann R, Bölker M, Schreier PH. 2005. A H_2O_2-producing glyoxal oxidase is required for filamentous growth and pathogenicity in *Ustilago maydis*. Mol Genet Genomics 272:639–650. https://doi.org/10.1007/s00438-004-1085-6.

(2) Park HD, Beeser AE, Clancy MJ, Cooper TG. 1996. The *S. cerevisiae* nitrogen starvation-induced Yvh1p and Ptp2p phosphatases play a role

in control of sporulation. Yeast 12:1135–1151. https://doi.org/10.1002/(sici)1097-0061(19960915)12:11<1135::aid-yea11>3.0.co;2-l

(3) Tan KC, Heazlewood JL, Millar AH, Thomson G, Oliver RP, Solomon PS. 2008. A signaling-regulated, short-chain dehydrogenase of *Stagonospora nodorum* regulates asexual development. Eukaryot Cell 7:1916–1929. https://doi.org/10.1128/EC.00237-08

(4) Hart RJ, Lawres L, Fritzen E, Ben Mamoun C, Aly AS. 2014. *Plasmodium yoelii* vitamin B5 pantothenate transporter candidate is essential for parasite transmission to the mosquito. Sci Rep 4:5665. https://doi.org/10.1038/srep05665.

(5) Hiatt KK, Ingram DA, Zhang Y, Bollag G, Clapp DW. 2001. Neurofibromin GTPase-activating protein-related domains restore normal growth in Nf1-/- cells. J Biol Chem 276: 7240–7245. https://doi.org/10.1074/jbc.M009202200.

(6) Wang CS, St Leger RJ. 2007. The MAD1 adhesin of *Metarhizium anisopliae* links adhesion with blastospore production and virulence to insects, and the MAD2 adhesin enables attachment to plants. Eukaryot Cell 6: 808–816. https://doi.org/10.1128/EC.00409-06.

(7) Tripathi A, Jain M, Chandra M, Parveen S, Yadav R, Collins BM, Maiti S, Datta S. 2020. EhC2B, a C2 domain-containing protein, promotes erythrophagocytosis in *Entamoeba histolytica* via actin nucleation. PLoS Pathog 16(5):e1008489. https://doi.org/10.1371/journal.ppat.1008489.

(8) Kabeche R, Baldissard S, Hammond J, Howard L, Moseley JB. 2011. The filament-forming protein Pil1 assembles linear eisosomes in fission yeast. Mol Biol Cell 22:4059–4067. https://doi.org/10.1091/mbc.E11-07-0605.

(9) Zeng GS, Wang YM, Wang Y. 2012. Cdc28-Cln3 phosphorylation of Sla1 regulates actin patch dynamics in different modes of fungal growth. Mol Biol Cell 23:3485–3497. https://doi.org/10.1091/mbc.E12-03-0231.

(10) Xie JL, Bohovych I, Wong E, Lambert JP, Gingras AC, Khalimonchuk O, Cowen LE, Leach MD. 2017. Ydj1 governs fungal morphogenesis and stress response, and facilitates mitochondrial protein import via Mas1 and Mas2. Microb Cell 4:342–36. https://doi.org/10.15698/mic2017.10.594.

(11) Li HY, Peng X, Wang YT, Cao SR, Xiong LP, Fan JJ, Wang YH, Zhuang SG, Yu XQ, Mao HP. 2016. Atg5-mediated autophagy

deficiency in proximal tubules promotes cell cycle G2/M arrest and renal fibrosis. Autophagy 12: 1472–1486. https://doi.org/10.1080/15548627.2016.1190071.

(12) Krüger A, Vowinckel J, Mülleder M, Grote P, Capuano F, Bluemlein K, Ralser M. 2013. Tpo1-mediated spermine and spermidine export controls cell cycle delay and times antioxidant protein expression during the oxidative stress response. EMBO Rep 14: 1113–1119. https://doi.org/10.1038/embor.2013.165.

(13) Cervantes Quintero KY, Padilla Guerrero IE, Torres Guzmán JC, Villa Martínez BG, Valencia Félix A, González Hernández GA. 2020. Members of the nitronate monooxygenase gene family from *Metarhizium brunneum* are induced during the process of infection to *Plutella xylostella*. Appl Microbiol Biotechnol 104: 2987–2997. https://doi.org/10.1007/s00253-020-10450-0.
